# An Investigation of Five Types of Personality Trait Continuity: A Two-Wave Longitudinal Study of Spanish Adolescents from Age 12 to Age 15

**DOI:** 10.3389/fpsyg.2016.00512

**Published:** 2016-04-18

**Authors:** Manuel I. Ibáñez, Ana M. Viruela, Laura Mezquita, Jorge Moya, Helena Villa, Laura Camacho, Generós Ortet

**Affiliations:** ^1^Department of Basic and Clinical Psychology and Psychobiology, Universitat Jaume ICastelló, Spain; ^2^Centre for Biomedical Research Network on Mental Health, Instituto de Salud Carlos IIIMadrid, Spain; ^3^Department of Pedagogy and Psychology, Universitat de LleidaLleida, Spain

**Keywords:** personality, development, stability, change, five-factor model

## Abstract

The present study investigated five types of personality trait continuity using two measurement waves of Spanish adolescents (*N* = 234). Personality traits were measured with the short form of the Junior Spanish NEO-PI-R (JS NEO-S) at ages 12 and 15. The results showed stability in the personality trait structure, as well as decreases in the mean levels of agreeableness and conscientiousness. The results also showed moderate rank-order consistency. Individual-level changes were more pronounced for neuroticism and conscientiousness. Approximately 90% of the participants showed ipsative consistency. The findings showed some personality trait changes occurred from age 12 to 15, but the changes were less marked than expected during this period of biological and social development. Our results also support the *disruption hypothesis*, as we found dips in conscientiousness and, to a lesser degree, agreeableness.

## Introduction

Personality is related to patterns of thoughts, feelings, and behaviors, which remain *relatively stable* during people's lifetime and distinguish one person from another. The present research will focus on the *relatively stable* part of the definition at a particular and fairly unexplored period, adolescence. The first term, *relative*, reflects the broadly assumed fact that personality is not perfectly stable through time, and its development implies some degree of change. Nowadays, the main research questions would refer to the extent of personality change in different stages of peoples' lives (Roberts et al., 2008; Soto and Tackett, 2015), and what are the causes and processes underlying these patterns of stability and change (Specht et al., 2014).

An additional difficulty in answering these questions is that the second term of the expression, *stability*, is indeed a multi-faceted construct. Five main types of stability/change have been proposed: structure consistency, rank-order consistency, mean-level change, ipsative consistency, and intra-individual differences in individual change (Roberts et al., 2008). Briefly, structural consistency refers to the persistence of correlational patterns among traits or dimensions over time, and it is considered to be the basic step in order to explore other types of stability. Rank-order consistency indicates whether the rank order of individuals in a certain trait is maintained over time. Mean-level stability is the extent to which absolute levels of personality scores change on average with time. Ipsative consistency and intra-individual differences in individual change have been the least explored types of personality continuity and change. Ipsative stability tracks the relative ordering of constructs (i.e., profile) within an individual over time, whereas individual change indicates the magnitude of the increase or decrease in any given trait exhibited by a person.

Extensive research on adult personality structure and development supports several key conclusions (Soto and Tackett, 2015). First, the Five-Factor Model (FFM) has become a consensus model that offers a useful descriptive taxonomy for most personality traits according to many personality psychologists (John et al., 2008). Second, the evidence of the structural continuity of the FFM from late adolescence to old age seems to be strong (Roberts et al., 2008). Third, and in terms of rank-order stability, the *cumulative-continuity principle* has been proposed, which refers to the fact that personality becomes increasingly stable across adulthood (for meta-analytic summaries see Roberts and DelVecchio, 2000; Ferguson, 2010). Finally, and in terms of mean-level development, studies consistently find that most people become more agreeable, conscientious, and emotionally stable with age, the so-called *maturity principle* (for a meta-analytic summary see Roberts et al., 2006).

Research on adolescent personality traits is more scarce than research on adult personality traits, so it is less clear if these conclusions may be applied to adolescent personality development. In relation to personality structure, substantial convergence between temperamental models and the FFM has been found in recent years (De Pauw and Mervielde, 2010), and there is compelling evidence that the FFM adequately represents the personality domain in adolescents (Goldberg, 2001; McCrae et al., 2002; Soto et al., 2008; Ortet et al., 2012). However, some differences have also appeared between youth and adult personality, although they seem more evident in childhood than in adolescence. For example, difficulties have been reported in identifying the openness/imagination dimension in younger samples (Mervielde et al., 1995; Lamb et al., 2002; De Fruyt et al., 2006; Soto et al., 2008). Moreover, possible additional temperamental factors have been described in childhood or early adolescence, such as irritability and activity (John et al., 1994; Lamb et al., 2002; Soto and Tackett, 2015). Finally, cross-sectional studies have found certain problems of structure differentiation, especially in younger samples and mainly within conscientiousness and agreeableness domains (Soto et al., 2008; Tackett et al., 2012). However, when structural continuity has been tested longitudinally, it has usually been found that the five-factor intercorrelations do not change substantially at a broad domain level (e.g., McCrae et al., 2002; De Fruyt et al., 2006), although some differences appeared at a facet level at younger ages (De Fruyt et al., 2006). Clearly, additional research is needed to further clarify the trait structural continuity during adolescence.

In reference to the mean-level stability of personality in adolescence, researchers have moved from an initial *maturation* vision (Roberts et al., 2006; Klimstra et al., 2012) to a *disruption* one in recent years. Soto et al. (2011), in a cross-sectional study of over a million participants, found that levels of agreeableness, conscientiousness, and openness declined from late childhood to early adolescence, then increased rapidly from late adolescence to early adulthood. Similar trends have been found in a meta-analysis using 14 cross-sectional and longitudinal studies (Denissen et al., 2013), and in posterior longitudinal studies (van den Akker et al., 2014; Göllner et al., 2016). These findings led to the *disruption hypothesis* in young personality development, which states that the biological, social, and psychological transitions from childhood to adolescence are accompanied by temporary dips in some aspects of personality maturity (Soto and Tackett, 2015).

Meta-analyses on rank-order stability (e.g., Roberts and DelVecchio, 2000; Ferguson, 2010) have indicated that stability indices (correlations) among personality traits increase with age from adolescence to adulthood. The data available from research works in adolescents have shown a similar picture, with lower rank-order stability during late childhood and early adolescence than during late adolescence and early adulthood (e.g., Pullmann et al., 2006; Akse et al., 2007; Klimstra et al., 2009).

Finally, individual stability types, such as individual and ipsative or profile changes, have been examined even less frequently than the above-mentioned types. The few previous findings obtained in adolescents have indicated that most individuals show no significant variations, and that openness is the dimension that presents more individual changes (McCrae et al., 2002; De Fruyt et al., 2006; Pullmann et al., 2006). Finally, the few previous findings obtained with ipsative stability indicated that changes occur in the profile personality configuration in fewer than 10% of adolescents (De Fruyt et al., 2006), while older teenagers present a more stable profile (Klimstra et al., 2012).

Only a handful of studies have been conducted on personality development across adolescence using the FFM. As far as we know, only one research work has studied up to five types of personality continuity in the same sample of young participants under the FFM framework (De Fruyt et al., 2006). Thus, longitudinal studies on the development of personality across adolescence will contribute to the understanding of personality continuity in this developmental stage.

The present study examined adolescents' five-personality dimensions development from age 12 to 15 through structural consistency, mean-level or normative change, rank-order consistency, structural consistency, individual differences in change, and ipsative consistency. According to the reviewed literature, the hypotheses were that we would find: (1) structural continuity of the five factors; (2) a decrease in agreeableness, conscientiousness, and openness according to the disruption hypothesis; (3) moderate levels of rank-order consistency; and (4) low levels of ipsative and individual change, with openness being the dimension with more intra-individual changes.

## Materials and methods

### Participants and procedure

The sample was composed of 371 secondary education students whose mean age was 12.03 years (*SD* = 0.60) and who answered the personality scales in 2004 (Time 1, T1). Of these, 234 (90 boys and 144 girls; mean age = 15.32, *SD* = 0.71) were re-assessed 3 years later in 2007 (Time 2, T2). The attrition analysis indicated that the participants who answered at both T1 and T2 were moderately more agreeable, *t*_(369)_ = 2.63, *p* = 0.009; and open to experience, *t*_(369)_ = 2.48, *p* = 0.014, than those who answered only at T1. However, there were no significant differences in the percentage of male and female participants or in their parents' socioeconomic status.

The participating students belonged to four high schools in the province of Castelló (east Spain). Four trained research assistants handed out the questionnaires, followed the standard instructions and encouraged respondents to give sincere answers. All the attending students voluntarily completed the questionnaires in the classroom and did not receive any compensation for their participation.

### Ethics

This study was carried out in accordance with the recommendations of the ethical committee from the Universitat Jaume I. Parents or legal tutors of the participants gave written informed consent in accordance with the Declaration of Helsinki.

### Measures

In this study we used a short form of the Junior Spanish NEO-PI-R (JS NEO-S; Ortet et al., 2007), which comprises 150 items, answered on a 5-point Likert-type scale ranging from *strongly disagree* to *strongly agree*. It assesses 30 specific traits or facets that define the five personality factors or domains: neuroticism, extraversion, openness to experience, agreeableness, and conscientiousness. JS NEO-S can be used in adolescents aged 12–18 years. In the present study the Cronbach alphas for the five personality domains in T1 were: neuroticism = 0.79, extraversion = 0.75, openness = 0.65, agreeableness = 0.74, and conscientiousness = 0.86, while in T2 were: neuroticism = 0.88, extraversion = 0.84, openness = 0.81, agreeableness = 0.78, and conscientiousness = 0.91.

### Data analyses

Different analyses were carried out according to the type of personality continuity studied. We examined two issues of s*tructural continuity* by means of two different analyses. First, we explored if intercorrelations among FFM dimensions changed from T1 to T2 following the procedure described by Robins et al. (2001) and De Fruyt et al. (2006). Thus, using structural equation modeling (SEM), we specified a baseline model that was a single-indicator latent variable model, with one latent variable associated with all 10 scores (five dimensions × two assessment occasions). This is a fully saturated model, with the variances of the latent variables fixed at 1 and the variances of the residuals fixed at 0. The correlations among the latent variables were freely estimated. In the second model, the correlations between all the pair-wise dimensions across the two assessment occasions were constrained to be equal. For instance, the correlation between neuroticism and conscientiousness at T1 was forced to equal the correlation between neuroticism and conscientiousness at T2. A significant difference in fit between these two models would indicate structural change.

Second, and in order to provide empirical criteria for evaluating the similarity of factor structures, we applied the Procrustes rotation following a similar procedure of Tackett et al. (2012). As the NEO-PI-R was initially developed in a USA sample in adults, we performed the Procrustes rotation toward the American normative structure of the adult NEO-PI-R (Costa and McCrae, 1992) to assess the factor replication of the obtained structure at T1 and T2 (for a similar procedure see also McCrae et al., 1996; Ortet et al., 2012). These comparisons offer empirical information about whether personality structures at T1 and at T2 differ from the adult personality structure. In addition, to examine the similarity in structure across time, we also obtained congruence coefficients between T1 and T2.

In order to carry out this rotation and obtain the congruence coefficients, we conducted two principal component analyses and varimax rotations on the JS NEO facets, first at T1 and after at T2. Afterward, we entered the loadings obtained from the 30 scales in our sample at T1 into the Procrustes syntax (see McCrae et al., 1996) to obtain the congruence coefficients between the five-factor structure at T1 and the original NEO-PI-R structure. This procedure was repeated with the loadings obtained at T2 in order to compare the factor structure at T2 with the American normative structure. Finally, both T1 and T2 30-scale loadings were entered into the program to calculate the congruence coefficients across time.

*Mean-level change* was assessed by comparing the mean personality scores between measurement times. *Rank-order stability* was assessed by the correlation between personality scores at each time point. *Individual differences in change* were computed using the RC index (Christensen and Mendoza, 1986; Jacobson and Truax, 1991; De Fruyt et al., 2006). This index calculates the probability of observing a difference in score equal to or greater than that obtained, assuming that no change occurred. This index accounts for the unreliability of measurement. Thus, it is a valuable technique for separating true personality change from change due to measurement error (Robins et al., 2001). The RC index is computed as RC = X_2_ − X_1_/S_diff_, where X_1_ signifies a person's score at Time 1, X_2_ denotes the same person's score at Time 2, and S_diff_ is the standard error of difference between the two personality scores. A detailed description of this method can be found in De Fruyt et al. (2006), p. 545.

Finally, two methods were employed for *ipsative consistency*. The first was the D^2^, D'^2^, D”^2^ indices proposed by Cronbach and Gleser (1953), which allowed us to obtain changes in the elevation (average level of scores), scatter (variability of scores), and shape (patterning of scores) of the individual profile of traits. The first index, D^2^, is sensitive to differences in elevation, scatter and shape, and quantifies the squared differences between traits on two assessment occasions. The second index, D′^2^, reflects differences only in scatter and shape, and quantifies the squared differences between trait profiles after each profile has been centered around its mean. The third index, D′^2^, is sensitive only to shape, and quantifies the squared differences between profiles after each profile has been standardized. They are computed as ${\text{D}}_{12}^{2}=\Sigma {\left({\text{x}}_{1}-{\text{x}}_{2}\right)}^{2}$; ${\text{D}}_{12}^{'2}={\text{D}}^{2}-5\Delta^{2}{\text{El}}_{12}$; ${\text{D}}_{12}^{\prime \prime 2}=\frac{{\text{D}}^{\prime 2}-\Delta^{2}\text{S}}{{\text{S}}_{1}{\text{S}}_{2}}$. Thus, D^2^ is the sum of the differences of the values of each score of the five dimensions between T1 and T2, Δ^2^El_12_is the squared difference of the mean scores, and S is the result of Ö5 (because we measured five personality variables) multiplied by standard deviation. In order to interpret these indices, a simulation of trait scores was performed on a sample of 50,000 individuals with identical levels of elevation, scatter and shape in a person's profile at the two measurement points, and the corresponding distributions were examined as reported in previous studies (Robins et al., 2001; De Fruyt et al., 2006). After obtaining the D^2^, D′^2^, D″^2^ indices, they were compared to the simulated value of the 95th percentiles, which would be the cutting point for the values of our samples. They indicate which individual profiles show significant changes in elevation, scatter, or shape. The second method of examining ipsative continuity was q-correlations (Ozer and Gjerde, 1989); that is, the within-person correlations across traits at T1 and T2. They are computed as: $r=\frac{\left({\text{score}}_{T1}-{\text{mean}}_{T1})({\text{score}}_{T2}-{\text{mean}}_{T2}\right)}{{\text{NSD}}_{T1}{\text{SD}}_{T2}}$. Correlation values of around 1 indicate that the pattern or configuration of traits is stable between the two measurement points. A value that is negative or around −1 suggests that the person shows an opposite personality profile between the two measurement points (e.g., De Fruyt et al., 2006; Klimstra et al., 2009, 2012).

## Results

### Structural consistency

We formally tested if intercorrelations among FFM dimensions changed from T1 to T2 using SEM. The base line model leads to a fully saturated model (CFI = 1.00). We reestimated the model after placing 10 pairwise equality constraints between paths at T1 and T2. A chi-squared difference test indicated that constraining the model did not lead to a significant reduction in fit, χ^2^Δ (*df* = 10) = 11.58, *p* = 0.314; CFI = 0.99, indicating that the saturated model did not fit better than the model with equal correlations. Therefore, we can conclude that the intercorrelations of the Big Five were structurally invariant across the two measurement points.

In addition, we performed the Procrustes rotation and obtained the factor congruence coefficients for each dimension. Thus, structure at T1 and the structure at T2 were rotated toward the adult North American structure of the NEO-PI-R, and structure at T1 was rotated toward the structure at T2 (see Table 1). According to McCrae et al. (1996) (see also Tackett et al., 2012) congruence coefficients >0.85 are higher than 95% of the rotations from random data, and factor congruence near 0.90 can be considered clearly replicated.

**Table 1:** **Factor congruence coefficients from T1 and T2 principal component analyses**.

	**T1: 12 y.o**.	**T2: 15 y.o**.	**T1 vs T2**
	**Spain vs. USA**	**Spain vs. USA**	
Neuroticism	0.91	0.92	0.91
Extraversion	0.89	0.95	0.91
Openness to experience	0.85	0.86	0.85
Agreeableness	0.92	0.88	0.94
Conscientiousness	0.92	0.94	0.91

*Congruence coefficients ≥86 are higher than 95% of the rotations from random data (McCrae et al., 1996)*.

When factor structures at T1 and T2 were compared to adult structure, the congruence coefficients were 0.85 and 0.86 for openness at T1 and T2, respectively, and ranged from 0.88 to 0.95 for the rest of traits (see Table 1). In addition, the congruence coefficients between T1 and T2 ranged between 0.90 and 0.94 for neuroticism, extraversion, agreeableness, and conscientiousness, but openness presented a coefficient of 0.85. Accordingly, our data indicates that Spanish adolescent structure at T1 and T2 closely approximates the American adult structure, and that the structure at T1 was clearly replicated at T2, although openness presented some replicability problems.

### Mean-level change

Table 2 presents the results of the mean comparisons made of the five personality dimensions between Times T1 and T2. The conscientiousness and, to a lesser extent, the agreeableness mean scores lowered, which may indicate a teenage rebellion pattern in this stage. No mean-level changes were found for neuroticism, extraversion, and openness.

**Table 2:** **Mean-level comparisons of the five dimensions at T1 and T2**.

	***M* T1**	***SD* T1**	***M* T2**	***SD* T2**	***t*-test *p***	**Cohen's *d***
Neuroticism	54.30	12.66	55.10	14.90	*ns*	0.06
Extraversion	77.74	11.21	78.90	13.06	*ns*	0.10
Openness to Experience	68.28	10.17	67.99	12.25	*ns*	−0.03
Agreeableness	79.75	10.41	77.51	10.73	<0.01	−0.21
Conscientiousness	82.10	12.73	70.52	15.61	<0.001	−0.81

*Cohen's d values of.20, 0.50, and 0.80 correspond to small, medium, and large effect sizes, respectively (Cohen, 1992)*.

### Rank-order consistency

The correlation indices of each personality dimension between T1 and T2 were 0.42 for neuroticism, 0.47 for extraversion, 0.49 for openness, 0.50 for agreeableness, and 0.45 for conscientiousness (see Table 3). The five index correlations were significant at *p* < 0.001. Thus, our results indicate that personality is relatively stable in adolescence.

**Table 3:** **Intercorrelations among the five dimensions**.

	**N**	**E**	**O**	**A**	**C**
Neuroticism (N)	0.42^***^	−0.20^**^	−0.06	−0.20^**^	−0.38^***^
Extraversion (E)	−0.18^*^	0.47^***^	0.19^*^	0.21^**^	0.11
Openness to experience (O)	−0.07	0.30^***^	0.49^***^	0.39^***^	0.20^**^
Agreeableness (A)	−0.19^**^	0.21^**^	0.21^**^	0.50^***^	0.36^***^
Conscientiousness (C)	−0.28^***^	0.21^**^	0.42^***^	0.11	0.45^***^

*The diagonal are the correlations between T1 and T2. Below the diagonal are the intercorrelations at T1. Above the diagonal are the intercorrelations at T2*.

* p < 0.05.

** p < 0.01.

*** *p < 0.001*.

### Individual differences in change

The RC indices of the five personality dimensions were computed (see Table 4). Lower percentages of participants were obtained for domains extraversion, openness, and agreeableness (fewer than 10%). Conversely, neuroticism (more than 20%) and conscientiousness (more than 30%) obtained the highest percentage of participants with significant RC indices. Furthermore, extraversion and agreeableness showed a balanced percentage of participants with lower and higher values for the RC indices, but conscientiousness presented the largest difference (more than a 30% decrease and less than a 2% increase).

**Table 4:** **Percentage of participants showing Reliable Change Index (RC)**.

	**No change %**	**% Decreased**	**% Increased**
Neuroticism	77.8	12.4	9.8
Extraversion	91.4	4.3	4.3
Openness to Experience	92.3	4.3	3.4
Agreeableness	92.3	6.0	1.7
Conscientiousness	65.4	32.9	1.7

### Ipsative consistency

Cronbach's and Gleser's D^2^, D′^2^, D″^2^ indices of the five domains were calculated. The results indicated low percentages of change in the elevation (*N* = 10, 4.3%), scatter (*N* = 9, 3.9%), or shape (*N* = 14, 6%) of individual profiles. Only 9.8% of the participants exhibited change in any of these three indices.

The q-correlations were also computed and the results revealed that the average was 0.49 (ranging from −0.52 to 0.80) and the median was 0.58. The Q_1_and Q_3_of the q-correlations distribution were 0.36 and 0.71, respectively. The 77.3% of the participants obtained q-correlations of over 0.30 and 6.4% of the participants obtained negative values (*r* < 0).

## Discussion

Traditionally, adolescence has been considered a difficult period of transition from childhood to adulthood, a time of “storm and stress.” Adolescent-typical behaviors such as conflict with parents, mood disruption, or risk behavior tend to occur during adolescence (Arnett, 1999), although there is a wide range of variability between individuals. Consequently, it has been proposed that a more comprehensive approach to adolescence should incorporate the role of temperament and personality characteristics (McAdams and Olson, 2010; Hollenstein and Lougheed, 2013).

Most studies on personality structure and development have been carried out in adults and, consequently, much less it is known about adolescent personality. The present research has studied five types of personality continuity in young adolescents who were longitudinally followed for 3 years. Demonstrating *structural stability* across T1 and T2 is an essential requirement before other forms of stability and change can be investigated (Roberts et al., 2008). Hence our results, as hypothesized, indicated that the five factors of personality were invariant across the two measurements. As also expected, agreeableness and conscientiousness significantly decreased for the *mean-level change*. The results also showed, as we predicted, *rank-order consistency* indices that Roberts and DelVecchio (2000) considered as moderate. In reference to individual types of stability/change, around 90% of participants also presented *ipsative consistency*, in line with previous findings (De Fruyt et al., 2006), whereas *individual differences in change* were greater for neuroticism and conscientiousness domains. Taken together, our findings support the view that personality presents some degree of variation during early adolescence as agreeableness and conscientiousness mean scores decreased, but it is more consistent over time than expected during this period of remarkable biological and social transformations (cf., Robins et al., 2001). However, we did not find the hypothesized mean decrease and individual changes in openness.

Very few longitudinal studies have examined structural personality change during adolescence (McCrae et al., 2002; De Fruyt et al., 2006). However, cross-sectional investigations indicate some differences between youth and late adolescence personality structure (Soto and Tackett, 2015). Our findings are in line with their results, which mean that no dramatic changes in the intercorrelations between domains, or in the structure of personality dimensions, occur during adolescence (McCrae et al., 2002; De Fruyt et al., 2006). However, some change trends previously described have been detected. For instance, the correlation between agreeableness and conscientiousness seems to present a slight decline from T1 to T2 (from 0.42 to 0.36), in line with the notion that a higher-order self-regulation trait would be more prominent at younger ages (Soto and Tackett, 2015). In relation to factor structure, a five-factor structure was clearly recognizable in the sample of the 12-year-old adolescents, in accordance with previous studies (McCrae et al., 2002; Ortet et al., 2012), and this structure did not change substantially at age of 15 years. Nonetheless some slight variations were detected, especially in openness, probably indicating that this trait is the most difficult to comprehend in this stage (Soto et al., 2008; Ortet et al., 2012).

In reference to normative continuity, we found significant changes in two of the five dimensions, a drop in agreeableness and conscientiousness, which may imply a rebellion and oppositional behavior pattern. A very similar pattern has been described in other cross-sectional and longitudinal studies (Soto et al., 2008; Denissen et al., 2013; van den Akker et al., 2014; Göllner et al., 2016), although some of these studies found that openness also tends to decline during this period. Thus, our results would support the *Disruption Hypothesis*, which posits that the transition from childhood to adolescence is accompanied by temporary dips in some aspects of personality maturity (Soto and Tackett, 2015).

For rank-order stability, previous meta-analyses (Ardelt, 2000; Roberts and DelVecchio, 2000; Ferguson, 2010) have stated that correlations in adolescence were around 0.40–0.50, which indicates moderate stability. Our results (between 0.42 and 0.50) replicate these findings and are in line with previous results for similar age ranges (e.g., McCrae et al., 2002; Akse et al., 2007).

Individual-level personality studies are also scarce, especially in adolescence. We found that very few adolescents obtained reliable higher or lower personality trait scores for individual differences in change. Most participants showed no significant changes (between 60 and 80%) for any personality dimension, which is in accordance with previous findings (McCrae et al., 2002; De Fruyt et al., 2006; Pullmann et al., 2006). Neuroticism and conscientiousness were the traits in which the minority of youngsters presented the most marked individual score changes. It seems that individual-level changes in one personality dimension, or more, are associated with particular life events (Lüdtke et al., 2011). For ipsative stability, fewer than 10% of the participants exhibited a change in the elevation, scatter or shape of individual personality profiles. The q-correlations also reflected a stable individual trait profile. Our results replicate some few previous studies, which were conducted on intra-individual consistency (De Fruyt et al., 2006; Klimstra et al., 2012), and support the idea that individual personality profiles are consistent in adolescence.

This study has two main limitations. The first is that there was a fairly substantial amount of attrition, which could affect the generalizability of our results. Those participants who were retained differed, although only slightly, in terms of mean personality scores from those who dropped out, as they were moderately more agreeable and open to experience. The second limitation is that this research only covered two time points during a 3-year follow-up period in adolescence. Three time points or more covering a longer period would enable us to examine non-linear development trajectories throughout adolescence.

In summary, the transition from childhood to adolescence has been traditionally viewed as a period of dramatic changes in personality development, probably more than in any other developmental stage. Our results, and taking into account previous studies, would contradict this vision in relation to the five factors of personality. Thus, adolescent personality development seems to be characterized by structural continuity of the FFM. In addition, although we found low to moderate changes in the other four stability/change types, they seem to be lower than those presented in other developmental stages, such as young adulthood (Roberts et al., 2006). In any case, studies that have examined the main five types of personality continuity in the same sample under the FFM framework are nearly inexistent, so further longitudinal investigations in adolescence that take into account structural, population, and individual levels of analysis in personality development are clearly necessary.

## Author contributions

GO and MI: designed the study, performed statistical analyses, and wrote the article. AV: designed the study, collected the data, performed statistical analyses, and wrote the article. LM, JM, HV, and LC: collected the data and performed statistical analyses.

## Funding

This research has been funded by grant PSI2008-05988 from the Spanish Ministry of Science.

### Conflict of interest statement

The authors declare that the research was conducted in the absence of any commercial or financial relationships that could be construed as a potential conflict of interest.
